# Emerging Nano‐/Microapproaches for Cancer Immunotherapy

**DOI:** 10.1002/advs.201801847

**Published:** 2019-01-13

**Authors:** Yu Mi, C. Tilden Hagan, Benjamin G. Vincent, Andrew Z. Wang

**Affiliations:** ^1^ Laboratory of Nano‐ and Translational Medicine Carolina Center for Cancer Nanotechnology Excellence Carolina Institute of Nanomedicine Lineberger Comprehensive Cancer Center Department of Radiation Oncology University of North Carolina at Chapel Hill Chapel Hill NC 27599 USA; ^2^ Lineberger Comprehensive Cancer Center Department of Microbiology & Immunology Curriculum in Bioinformatics and Computational Biology Division of Hematology/Oncology Department of Medicine University of North Carolina at Chapel Hill Chapel Hill NC 27599 USA

**Keywords:** adoptive immunotherapy, cancer immunotherapy, cancer vaccines, checkpoint blockades, combination immunotherapy, nanoparticles

## Abstract

Cancer immunotherapy has achieved remarkable clinical efficacy through recent advances such as chimeric antigen receptor‐T cell (CAR‐T) therapy, immune checkpoint blockade (ICB) therapy, and neoantigen vaccines. However, application of immunotherapy in a clinical setting has been limited by low durable response rates and immune‐related adverse events. The rapid development of nano‐/microtechnologies in the past decade provides potential strategies to improve cancer immunotherapy. Advances of nano‐/microparticles such as virus‐like size, high surface to volume ratio, and modifiable surfaces for precise targeting of specific cell types can be exploited in the design of cancer vaccines and delivery of immunomodulators. Here, the emerging nano‐/microapproaches in the field of cancer vaccines, immune checkpoint blockade, and adoptive or indirect immunotherapies are summarized. How nano‐/microparticles improve the efficacy of these therapies, relevant immunological mechanisms, and how nano‐/microparticle methods are able to accelerate the clinical translation of cancer immunotherapy are explored.

## 1 Introduction

### 1.1 Current Progress in Cancer Immunotherapy

**Figure 1:**
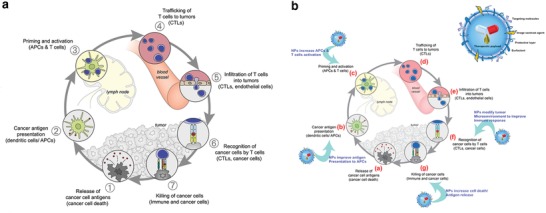
a) The cancer immunity cycle. Reproduced with permission.^1^ Copyright 2013, Cell Press. b) Depiction of the complex pathway involved in cancer immunotherapy. Nanoparticle delivery vehicles can play a role at multiple points along this pathway. Reproduced with permission.^86^ Copyright 2017, American Chemical Society.

T‐cell‐mediated cancer immunity includes numerous sequential steps involving: release of cancer cell antigens, delivery of cancer antigens to antigen presenting cells (APCs), successful presentation of antigen to T cells, priming and activation of APCs and T cells, trafficking and infiltrating of antigen specific T cells into tumors, recognition of cancer cells by T cells, and overcoming immunosuppression in the tumor microenvironment to affect cell‐mediated cytotoxicity, a process known as the “Cancer Immunity Cycle” (**Figure** **1**a).^1^ Based on this understanding, several novel strategies have been developed to enhance cytotoxic T‐cell activation and have achieved impressive clinical outcomes.^2^, ^3^, ^4^, ^5^, ^6^, ^7^, ^8^, ^9^ One promising strategy is chimeric antigen receptor‐T cell (CAR‐T) therapy.^10^, ^11^, ^12^, ^13^, ^14^ In CAR‐T therapy, a patient's own T cells are isolated and genetically modified via a viral vector to express chimeric antigen receptors (CARs), and infused back into the same patient. Chimeric antigen receptors contain an extracellular antigen‐binding domain, usually a single chain fragment derived from the variable region of an antibody, and a linker to an intracellular signaling domain, such as CD3ζ. Once the extracellular domain of CAR‐T cells recognizes a specific antigen, it triggers the signaling domain for T‐cell activation, proliferation, and elimination of target cells. In 2017, CAR‐T therapy targeting the B‐cell antigen CD19 was approved by the US Food and Drug Administration (FDA) for childhood acute lymphoblastic leukemia, based on an overall remission rate of 82.5%.^15^, ^16^ However, the current efficacy of CAR‐T therapy in other hematological malignancies and solid tumors is inadequate,^17^, ^18^ and safety issues during manufacturing require additional investigation.^19^, ^20^

In addition to CAR‐T therapy, immune checkpoint blockade (ICB) has generated exciting clinical results. Instead of direct stimulation of cytotoxic T cells, ICB targets and inhibits tumor‐mediated immunosuppression.^21^, ^22^, ^23^ T‐cell activation and function are regulated by both costimulatory and inhibitory ligand/receptor interactions between T cells and APCs, including both dendritic cells and tumor cells. These receptor/ligand pairs for immune regulation are called immune checkpoints. Tumors rely on taking advantage of certain immune checkpoint pathways to escape from the host immune response. As receptor/ligand interactions can be interrupted by antibodies or recombinant forms of ligands or receptors, inhibitory agents have been engineered to target and block immune checkpoints, overcoming tumor immune resistance. Representative immune checkpoint inhibitors include monoclonal antibodies against cytotoxic T‐lymphocyte antigen‐4 (CTLA‐4), programmed cell death protein 1 (PD‐1) and its paired ligand PD‐L1. CTLA‐4 is a receptor expressed on T cells that downregulates T‐cell activation at early stages and competes with the homologous stimulating receptor CD28 in binding CD80 and CD86 on APCs. While CTLA‐4 on CD4^+^ T helper cells downregulates their activity, it conversely upregulates regulatory T‐cell (Treg) activity, weakening the immune response in both cases. Blocking the signal from CTLA‐4 receptors can increase T‐cell activation and boost the antitumor immune response. Ipilimumab, a CTLA‐4 antibody, has been shown to improve overall survival and the 5 year rate of recurrence‐free survival in patients with advanced melanoma and was the first monoclonal antibody approved by FDA in 2011.^24^, ^25^ Another commonly targeted immune checkpoint is the PD‐1/PD‐L1 receptor/ligand pair.^23^, ^26^, ^27^, ^28^ The PD‐L1 ligand is expressed on many tissues to limit autoimmunity, but it is overexpressed on tumors and recognized as a major immune resistance mechanism in the tumor microenvironment. PD‐1 is a T‐cell receptor (TCR) that limits effector T‐cell activity but causes Treg proliferation, both of which lead to immunosuppression when activated within tumors. The PD‐1/PD‐L1 inhibitors, including pembrolizumab (anti‐PD‐1), nivolumab (anti‐PD‐1), and atezolizumab (anti‐PD‐L1), have been approved by the FDA with response rate of up to 40–50%^29^ for several cancers such as advanced melanoma, non‐small‐cell lung cancer (NSCLC), head and neck squamous cell carcinoma, advanced urothelial carcinoma, advanced kidney cancer, advanced liver cancer, advanced bladder cancer, colorectal cancer, and classical Hodgkin lymphoma.^30^ In addition to CTLA‐4 and PD‐1/PD‐L1, several other inhibitory checkpoints are under investigation, including B7 family inhibitory ligands B7‐H3 and B7‐H4, lymphocyte activation gene 3 (LAG3), T‐cell immunoglobulin 3 (TIM3), and T cell‐immunoglobulin and ITIM domain (TIGIT). Despite the promising results of ICB therapy, issues still exist. For example, ICB monotherapy can lead to resistance and lower the durable response rate.^31^, ^32^ Recent clinical results suggest that combination immunotherapy can further improve the efficacy of ICB, such as combining anti‐PD‐1 with anti‐CTLA‐4.^33^, ^34^, ^35^, ^36^, ^37^ However, such combination suffers from suboptimal activation and increases autoimmune toxicity.^38^

Another attractive strategy for enhancing the anticancer immune response is vaccination with tumor‐specific antigens.^39^, ^40^, ^41^, ^42^ Cancer cells express numerous mutations and mutant protein sequences that can be processed into short peptides by APCs. They are then presented on the APC‐cell surface by major histocompatiblility complex (MHC) molecules and can be recognized by T cells as foreign antigens. Some of these antigens are tumor‐specific and not expressed on normal cells, called *neoantigens*. Compared to tumor‐associated self‐antigens, neoantigens can bind to TCRs with higher affinity and induce more robust T‐cell responses. As they are recognized as nonself by the host immune system, they are less likely to induce central and peripheral tolerance, and as they are absent from normal cells, they are less likely to be targets for autoimmunity. Therefore, vaccination with neoantigens has become one of the most intriguing strategies for cancer immunotherapy.^43^, ^44^, ^45^ Neoantigens can be identified by comparing tumor DNA sequences with those isolated from normal host cells. Because most neoantigens are predicted from unique mutations and not shared among patients, vaccination with neoantigens must be personalized. Recent studies have shown approaches to realize personalized neoantigen vaccines with promising results in patients.^46^, ^47^, ^48^ Typically, tumor biopsies and healthy cell DNA are taken from a patient for identification of neoantigens via whole‐exome sequencing. Mutations are ranked according to the binding affinity to MHC class I and/or class II. The selected mutations are synthesized into DNA, RNA, or peptides and formulated with vaccine adjuvant to produce personalized vaccines. Patients receiving these neoantigen vaccines can develop neoantigen‐specific T cells for tumor killing. Some phase I clinical studies showed that the cumulative rate of metastatic events was significantly reduced after the start of vaccination, resulting in sustained progression‐free survival.^46^, ^47^ Although current results demonstrate the feasibility of neoantigen vaccination, limitations remain. First, the efficiency of neoantigens greatly relies on their cross‐presentation by APCs, which is critical in increasing the immunogenicity of neoantigens.^43^, ^45^, ^48^ Second, the diversity of tumor subpopulations makes it impossible to eradicate most tumors with immune responses to a single antigen. The delivery of a combination of neoantigens should be utilized to maximize anticancer efficiency against all tumor subpopulations. Third, the presence of neoantigens, even when effectively presented, is not enough for cancer immunity due to immunosuppression and resistance. Combination therapy is necessary to overcome the immunosuppressive tumor microenvironment and treatment‐induced resistance.

### 1.2 Nanotechnology and Nanomedicine

Nanotechnology is widely used in drug delivery to improve the efficacy of chemo‐/radiotherapy or photodynamic therapy due to the unique physiological properties of nanoparticles.^49^, ^50^, ^51^, ^52^, ^53^, ^54^, ^55^ The purpose of drug delivery is to enlarge the therapeutic window by increasing the concentration of therapeutics in tumors and decreasing their biodistribution in normal tissues. To achieve this, it is important to prolong the systematic circulation of antitumor agents and improve their targeting of tumor cells. Nanoformulations of drugs show several advantages in expanding therapeutic windows. First, the increased size of nanoparticles avoids rapid renal elimination as normally occurs with small molecules. Additionally, PEGylation of nanoparticles avoids opsonization, where they are cleared via the mononuclear phagocytic system. Moreover, nanoparticles can enhance drug accumulation within the tumor through passive and active targeting. The abnormal vasculature in tumors elicits a greater infiltration of nanoparticles than in normal tissue. Meanwhile, the ineffectiveness of lymphatic drainage within tumors increases the accumulation and retention of these infiltrating nanoparticles inside tumors, a phenomenon called the enhanced permeability and retention (EPR) effect. Nanoparticles can also actively target cancer cells through a ligand/receptor manner, binding of surface moieties that would likely be more highly expressed on tumor cells. Over the past few decades, a variety of studies have focused on improving nanoparticle's anticancer efficacy through the development of novel biomaterials; optimizing the size, shape and surface coating of nanoparticles; or screening targeting ligands with high binding affinity to cancer cells. Most of the attempts achieved promising results in animal models, however only a few nanoformulations have shown significant improvement in patients,^56^, ^57^, ^58^ indicating a limitation of the EPR effect.

### 1.3 Nanomedicine and Cancer Immunotherapy

Along with advances in immunology, researchers began to explore the potential of nanotechnology for improving cancer immunotherapy.^59^, ^60^, ^61^, ^62^, ^63^, ^64^, ^65^, ^66^, ^67^, ^68^ Unlike conventional nanomedicine that directly targets cancer cells, nanoparticles designed for cancer immunotherapy also target lymphocytes or APCs in circulating blood or lymphoid tissues.^69^, ^70^, ^71^, ^72^, ^73^ Stimulated immune cells can then traffic along chemokine gradients to tumors and directly attack tumor cells. It has been shown that nanoparticle‐carrying T cells or natural killer cells can concentrate drugs in tumors more than nanoparticles alone,^74^, ^75^, ^76^, ^77^ indicating that stimulating immune cells can be more effective than direct delivery of anticancer drugs to tumors using nanoparticles. Moreover, previous drug delivery studies have shown that in order to achieve total tumor eradication, an extremely high concentration of cytotoxic agents need to be delivered into tumors, which is difficult to achieve, even with the EPR effect. However, in cancer immunotherapy, a much lower concentration of immunomodulators is required for a robust and durable anticancer immune response, as activated immune cells can proliferate and expand with a memory effect.

This strategy in using nano/microparticles for cancer immunotherapy overcomes several limitations in previous nanomedicine. First, nanoparticles directly targeting tumor cells should be able to avoid opsonization and minimize unwanted uptake by phagocytes. However, due to their virus‐like size, they are easily taken up by macrophages or other APCs, which leads to a decreased accumulation in tumors. When nanoparticles are incorporated with tumor antigens, these effects can alternatively become an advantage for extending antigen exposure to APCs. In fact, early studies have developed virus‐based cancer vaccines with impressive anticancer effects.^78^, ^79^, ^80^ Major concerns for viral vaccines are their possible toxicity and safety issues. Synthetic nanoparticles have similar size distribution and immunogenicity as viral vaccines, with far fewer safety issues. Currently, a number of studies have shown that nanoparticle‐bound antigens can elicit greater immune responses than free antigens. Second, due to the high surface area to volume ratio of nanoparticles, they are able to enhance cross‐presentation of tumor antigens to APCs. An efficient activation of TCRs needs sustained stimulation by antigens and assembly of large receptor clusters at the T‐cell surface. During this process, the density of peptide‐major histocompatibility complexes (pMHCs) plays an important role for agonistic activity. One way to achieve a high pMHC density is by coating pMHC on nanoparticles. The high density of pMHC on the NP surface expedites the re‐engagement of dissociated pMHC, thus delaying TCR internalization and prolonging the half‐life of individual TCR‐pMHC interactions. The prolonged interaction increased sustained assembly of large TCR microclusters and further amplified the duration and magnitude of TCR signaling.^81^, ^82^, ^83^ Third, nanoparticles can also actively target specific subsets of immune cells by integrating antibodies or other targeting ligands on their surface. Potential targeting receptors include lineage markers such as CD8 or functional markers such as PD‐1. While nonspecific immune stimulation can cause autoimmune toxicity such as colitis, hepatitis and pneumonitis,^84^ targeted delivery of immunomodulators by nanoparticles can better enhance T‐cell function with decreased toxicity over systemic administration of free immunomodulators.^85^ Last, nanoparticles can be an ideal tool to incorporate multiple immunomodulators for simultaneous costimulation of immune cells. Effective activation of APCs or T cells usually involves stimulating multiple signaling pathways at the same time. However, administration of free immunomodulators results in some immune cells only encountering one immunomodulator or the other, some sequentially encountering both but at different times so there is no costimulation, and only a subset of immune cells that encounter both simultaneously providing the desired costimulatory effects. Combining immunomodulators within or on nanoparticles can provide simultaneous presentation to avoid this type of suboptimal activation.

The typical immunity cycle displayed in figure 1b demonstrates the numerous possible stages at which nanoparticles can improve immune responses (Figure 1b).^86^ For each specific stage which can be affected, optimization of nano‐/microparticles must be re‐evaluated to maximize immunotherapy efficacy, such as increasing the immunogenicity of nanoparticles, efficiently targeting immune cells, cross‐presenting tumor antigens to APCs, and codelivering immunomodulators to lymphoid organs. In this review, we will discuss how nano‐/microtechnologies boost the host immune response by summarizing and analyzing recent nano‐/microapproaches for cancer immunotherapy.

## 2 Nanotechnology for Cancer Vaccine Development

### 2.1 Delivery of Protein Antigens

**Figure 2:**
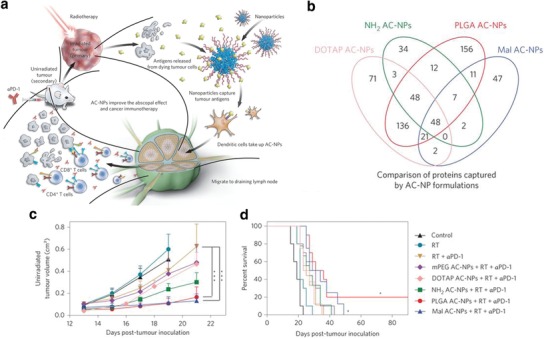
a) Schematic depiction of utilizing AC‐NPs to improve cancer immunotherapy. b) Comparison of proteins bound to AC‐NPs with different surface chemistries. c) Average tumor‐growth curves of unirradiated (secondary) tumors in individual mice treated with immunotherapy and AC‐NP formulations. d) Survival curves of the mice treated with immunotherapy and AC‐NP formulations. Reproduced with permission.^88^ Copyright 2017, Nature Publishing Group.

The surface chemistry of nanoparticles can be engineered such that they can capture tumor‐derived protein antigens. The antigen‐capturing nanoparticles further enhance the exposure of antigens to antigen‐presenting cells. Wang's group has shown that using antigen‐capturing nanoparticles (AC‐NPs) enhances the abscopal effect (**Figure** **2**), a phenomenon whereby local radiotherapy induces a systematic immune response and the regression of metastatic lesions. Radiation produces pro‐inflammatory proteins and increases exposure of immune cells to cancer‐specific antigens released after radiotherapy‐induced cancer cell death.^87^ Wang and co‐workers developed several AC‐NPs to bind these tumor‐derived protein antigens through a variety of mechanisms induced by changes in surface chemistry, such as noncovalent hydrophobic‐hydrophobic interactions, ionic interactions and covalent interactions. These antigen‐bound AC‐NPs were successfully trafficked to nearby tumor‐draining lymph nodes by dendritic‐cell‐mediated transport. When combined with anti‐PD‐1 treatment, AC‐NPs generated up to a 20% cure rate compared to 0% without AC‐NPs in a B16‐F10 melanoma model. This enhanced efficacy was due to an improved immune response, the mechanism for which was demonstrated to be from AC‐NPs inducing an expansion of CD8+ cytotoxic T cells and increasing both CD4+T/Treg and CD8+T/Treg ratios (Treg, regulatory T cells).^88^ Liu and co‐workers improved radio‐immunotherapy by developing a sodium alginate formulation containing ^131^I‐labeled catalase. The soluble alginate can transform into hydrogel in tumor in the presence of Ca^2+^. Meanwhile, the ^131^I‐labeled catalase enables hypoxia‐relieved radioisotope therapy. They showed that their radio‐enhanced alginate formulation, when loading with cytosine‐guanine oligodeoxynucleotide (CpG ODN), triggered strong systemic antitumor immune responses. Combining their system with CTLA‐4 checkpoint blockade led to synergistic effect in eliminating distant metastatic tumors and eliciting long‐term immune memory effect in mouse model.^89^

In another study, researchers showed how the nature of antigen association on nanoparticles (adsorption vs encapsulation) and the type of surfactants (poly(vinyl alcohol) (PVA) or PF127) affected the activation of DCs. Chitosan‐mixed poly(lactide‐*co*‐glycolide) (PLGA) or poly(lactide‐*co*‐glycolide)‐block‐poly(ethylene glycol) (PLGA‐b‐PEG) nanoparticles were developed to either absorb ovalbumin (OVA) antigens or encapsulate them. These nanoparticles were stabilized by either PVA or PF127. The results revealed that antigen‐adsorbed NPs induced higher expression of MHC II on DCs, while antigen‐encapsulated NPs induced higher expression of MHC I. Moreover, DCs expressed higher levels of CD86 when they were activated by PVA‐coated NPs instead of PF127‐coated NPs. Codelivery of OVA and CpG with PVA‐coated, antigen‐encapsulated NPs induced the best antigen‐specific T‐cell response.^90^

Other attempts to deliver protein antigens include developing a DC‐targeted nanoparticle composed of mannose‐modified alginate^91^ or chitosan,^92^ a synthetic vaccine nanoparticle made of aminated γ‐PGA‐cholesterol conjugate,^93^ a nanogel composed of a poly(hydroxyethyl methacrylate) (pHEMA) backbone and a functionalized hydrophobic side chain of pyridine,^94^ or a photosensitizer‐conjugated polyethyleneimine (PEI) nanoparticle.^95^

### 2.2 Delivery of Peptide Antigens

Peptide‐based cancer vaccines have been widely investigated because of their robust safety profile and ease of manufacturing. However, their clinical efficacy is limited due to rapid renal clearance and enzymatic biodegradation. More importantly, antigens presented by immature dendritic cells in the absence of immune adjuvants such as toll‐like receptor (TLR) agonists (e.g., CpG ODN, Poly I:C), stimulator of interferon gene (STING) agonists, or cytokines (e.g., IL‐2, GM‐CSF) will induce tolerance rather than stimulate an immune reaction.^96^ In clinical trials, antigens are administered in depot‐forming water‐in‐oil emulsions such as incomplete Freund's adjuvant. However, administration of free antigens within adjuvants will lead to inefficient colocalization into draining lymph nodes, where the antigen presentation occurs.

**Figure 3:**
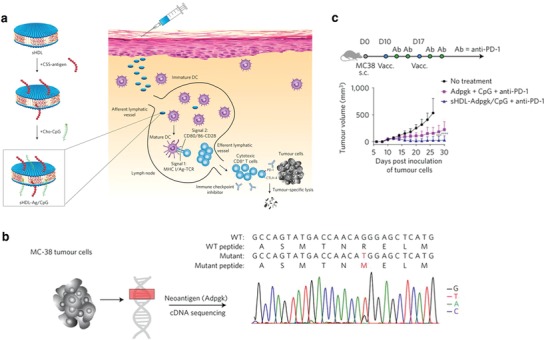
a) Design of sHDL nanodisc platform for personalized cancer vaccines. b) Mutation of Adpgk in MC‐38 murine colon adenocarcinoma cells was confirmed by sequencing cDNA of Adpgk. c) Treatment of MC‐38 tumor‐bearing C57BL/6 mice. Reproduced with permission.^97^ Copyright 2017, Nature Publishing Group.

As an alternative to free antigen administration, delivering peptide antigens attached to nanoparticles offers several advantages. First, nanoparticles are able to protect the peptides from peptidases during transportation, increasing peptide circulation time and total delivery. Second, nanoparticles have a virus‐like size distribution which causes them to be recognized and taken up by APCs, leading to a greater accumulation of antigens in lymphoid tissues. The accumulated antigens can induce cross‐presentation on dendritic cells and stimulate a strong cytotoxic T‐cell response. Third, nanoparticles can codeliver antigens and immune adjuvants simultaneously to avoid immune tolerance. In one case a synthetic high‐density lipoprotein nanodisc composed of an apolipoprotein A1 mimic peptide and phospholipids was manufactured to codeliver CpG and tumor peptide neoantigens (**Figure** **3**). The codelivery nanodisc vaccine elicited up to 47‐fold greater frequencies of neoantigen‐specific CD8+ cytotoxic T cells than free CpG plus peptide neoantigens. When combined with anti‐PD‐1 and anti‐CTLA‐4, the nanodisc vaccine led to complete tumor regression in ≈88% of mice bearing MC‐38 tumors and ≈90% of mice bearing B16F10 tumors, compared to only ≈25% and ≈38% of mice having complete tumor regression after treatment with free CpG plus peptide neoantigen.^97^ The same group also reported delivering immunogenic chemotherapeutics^98^, ^99^, ^100^, ^101^ using the same type of nanodisc. In this scenario, doxorubicin‐loaded nanodiscs potentiated a cytotoxic T‐cell response and increased the therapeutic effect of anti‐PD‐1 checkpoint blockade therapy.^102^ The nanodisc was also used to codeliver CpG and TLR4 agonists and improved antigen‐specific CD8+ T‐cell response by eightfold.^103^ Furthermore, Zhang and co‐workers developed SR‐B1‐targeting liposomes for delivering a fusion tumor peptide antigen. The authors demonstrated that the liposomes efficiently trafficked to lymph nodes and targeted mature DCs through SR‐B1‐medicated uptake.^104^ In another example, a TLR7 and TLR8 bi‐specific agonist was encapsulated into PLGA nanoparticles. The nanoadjuvant induced high‐levels of excretion of inflammatory cytokines and promoted DC activation. Combining the nanoadjuvants with OVA peptide or tumor cell lysate triggered a robust antigen‐specific immune response and reduced tumor growth and metastasis.^105^ Researchers also developed pyruvate dehydrogenase E2 protein nanoparticles for codelivery of CpG and gp100.^106^ Chen et al. integrated TLR7 agonist (imiquimod) into PLGA nanoparticles and coencapsulated photothermal agent (indocyanine green) for combination therapy. The nanoparticles showed vaccine‐like functions. When combining with CTLA‐4 checkpoint inhibitors, they showed strong immune response and memory effect for various types of tumor models.^107^

**Figure 4:**
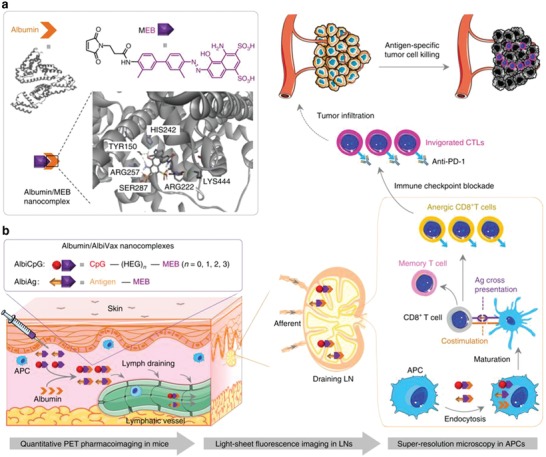
Schematic of albumin/AlbiVax nanocomplexes for efficient vaccine delivery and combination cancer immunotherapy. Reproduced with permission.^108^ Copyright 2017, Nature Publishing Group.

Albumin‐drug conjugates are commonly used protein nanocarriers for drug delivery. Abraxane is an FDA approved albumin‐paclitaxel conjugate for metastatic breast cancer, advanced non‐small‐cell lung cancer and metastatic pancreatic cancer. Compared with molecular drugs, albumin‐drug conjugates provide prolonged half‐life in blood and enhanced antitumor efficacy. Recently, researchers have designed an albumin nanovaccine for cancer immunotherapy (**Figure** **4**). Due to the viral‐like size effect, the albumin nanovaccine potentially drains to lymph nodes and provides enhanced exposure of antigens and adjuvants to APCs. To avoid complications during manufacturing, researchers conjugated peptide antigen to Evans blue that can bind to albumin and self‐assemble into a nanovaccine in vivo. The albumin nanovaccine was demonstrated to elicit 10 times more frequent peripheral antigen‐specific CD8+ cytotoxic T lymphocytes with immune memory, compared to a vaccine made of incomplete Freund's adjuvant (IFA) emulsifier. Its tumor inhibitory effect was significantly better than the IFA vaccine or free peptide antigens plus CpG.^108^ In another study, researchers synthesized an amphiphilic form of CpG by conjugating a diacyl lipid to the 5′ terminus of CpG ODN. The diacyl lipid can bind to albumin in blood to facilitate the accumulation of CpG in draining lymph nodes. The authors showed an enhanced accumulation of albumin‐binding CpG under a radiation‐enhanced EPR effect, which led to decreased off‐target effects in the liver and kidneys, and increased activation of CD8+ T cells and monocytes/macrophages in tumors.^109^

**Figure 5:**
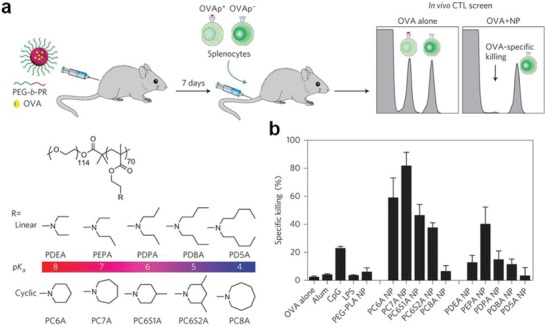
a) Schematic of the carboxy fluorescein succinimidyl ester (CFSE) method to screen for polymer structures that generate a strong OVA‐specific CTL response. b) Quantitative comparison of OVA‐specific CTL responses in different NP groups, identifying the PC7A NP as the best candidate. Reproduced with permission.^113^ Copyright 2017, Nature Publishing Group.

**Figure 6:**
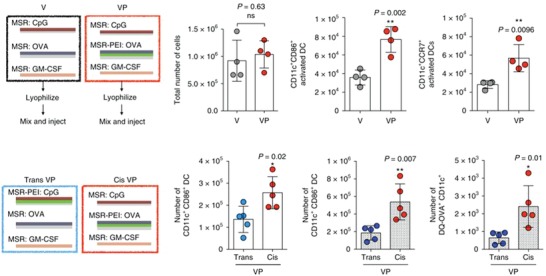
MSR–PEI vaccine enhances DC activation and trafficking in situ. Reproduced with permission.^114^ Copyright 2018, Nature Publishing Group.

Beyond codelivery of antigens and adjuvants, nanoparticles composed of certain biomaterials themselves can be effective immune adjuvants.^110^, ^111^, ^112^ Gao's lab developed a library of ultra‐pH‐sensitive (UPS) NPs (20–50 nm in diameter), which consist of copolymers containing tertiary amines with linear or cyclic side chains (**Figure** **5**). Through in vitro screening, they showed that PC7A nanoparticles induced the strongest cytotoxic T lymphocyte response through stimulating the STING‐type I IFN pathway. Delivery of tumor‐associated antigens or neoantigens with PC7A nanoparticles showed significant delay in tumor growth. The immune stimulatory effect of PC7A was stronger than poly (I:C) or CpG in a B16‐OVA melanoma mouse model. When combining PC7A nanoparticles with anti‐PD‐1 checkpoint blockade therapy, they showed synergistic tumor inhibitory effects in TC‐1 tumor models.^113^ In another study, PEI was utilized as an adjuvant to enhance antigen immunogenicity by incorporating PEI onto a mesoporous silica microrod (MSR) (**Figure** **6**). The presence of PEI increased the expression of CD86 and MHC‐II, and the production of tumor necrosis factor α (TNF‐α), interleukin‐6 (IL‐6) and IL‐1β on bone‐marrow‐derived dendritic cells (BMDCs). The MSR‐PEI was used to combine CpG, GM‐CSF, and neoantigens as vaccines for cancer treatment. The vaccine showed efficient tumor eradication in several tumor models with enrichment of IFN‐γ+, TNF‐α+, and granzyme B+ tumor‐infiltrating lymphocytes. In contrast, MSR vaccine without PEI did not generate enhanced tumor‐infiltrating lymphocytes, indicating the immunogenicity of PEI in cancer vaccines.^114^ In another study, researchers formed nanoadjuvants with nucleic acid‐based nanotechnology, where CpG and stat3 short hairpin RNA (shRNA) were incorporated into a single nanostructure. The nucleic acid nanoparticle was further shrunk and stabilized by PEG‐grafted polypeptide for codelivery of peptide neoantigens. This nanovaccine elicited eightfold more frequent neoantigen‐specific peripheral CD8+ T cells than CpG alone, and induced significant tumor regression.^115^ Researchers also exploited curdlan and mannan for their DC activation ability. Curdlan and mannan can be recognized by surface receptors of DCs and promote their secretion of cytokines. Therefore, researchers synthesized a pH‐sensitive derivative of curdlan and mannan by conjugating them with 3‐methylglutaric anhydride. Delivery of tumor antigens with these polysaccharide‐modified liposomes led to a stronger immune response in DCs than with dextran‐modified liposomes.^116^

**Figure 7:**
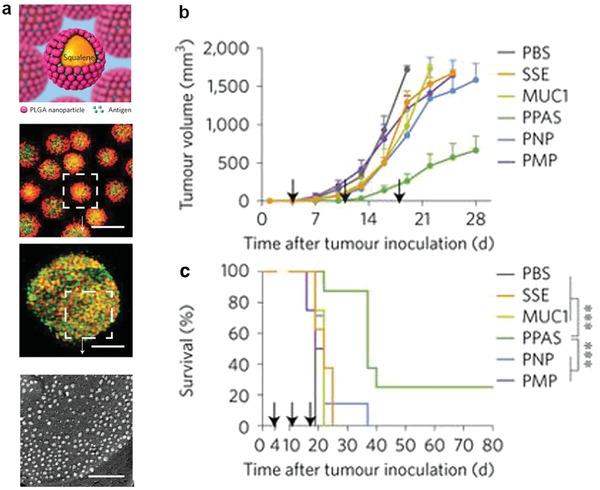
a) Schematic representation and characterization of PPAS. b) Average tumor growth curves and c) survival rate of the vaccinated mice showing that PPAS significantly induced tumor regression for MUC1 peptide therapeutic vaccination. Reproduced with permission.^117^ Copyright 2018, Nature Publishing Group.

In conventional drug delivery, a size range from 10 to 200 nm is generally optimal for the EPR effect. However, when designing a cancer vaccine, size is no longer restricted to the nanorange. Microparticles were also used to increase the immunogenicity of a cancer vaccine. For example, a Pickering emulsion is a particle‐stabilized emulsion that retains force‐dependent deformability and lateral mobility for presented antigens (**Figure** **7**). It can enhance the contact area and multivalent interaction between a vaccine and APCs via increasing the membrane dynamic curvature and the lateral diffusion of the absorbed antigens. By using a PLGA‐nanoparticle‐stabilized Pickering emulsion adjuvant system (PPAS), researchers showed that it elicited more efficient stimulation of APCs than solid individual polymeric nano‐/microparticles. A Pickering emulsion loaded with tumor‐associated MUC1‐peptide antigens induced greater survival rate than other FDA approved adjuvants such as alum, MF59 and AS04.^117^ Another example of a microvaccine is a recombinant Saccharomyces cerevisiae (YCP) coated with an indoleamine 2,3‐dioxygenase (IDO) siRNA‐loaded nanoparticle and tyrosinase‐related protein 2 (Trp2). The microparticle was positively charged and had an approximate size of 5 um. It successfully inhibited IDO expression in DCs and promoted Trp2‐specific CD8+ T‐cell responses.^118^ In addition to spherical microparticles, mesoporous silicon microdiscs were developed to codeliver TLR agonists (CpG and monophosphoryl lipid A (MPLA)) and TRP2 peptide antigens.^119^

### 2.3 Delivery of Nucleic Acid Antigens

**Figure 8:**
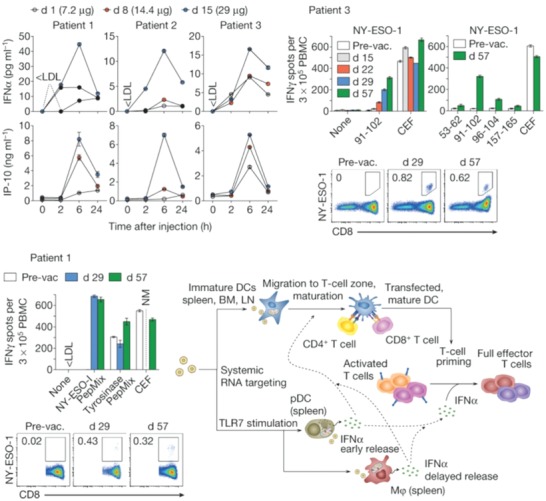
Clinically administered RNA‐LPX vaccines dose‐dependently induce systemic INFα and de novo priming and amplification of T cells against vaccine antigens. Reproduced with permission.^123^ Copyright 2016, Nature Publishing Group.

Delivery of nucleic acid‐based tumor antigens by nanoparticles can protect them from nucleases and increase their drainage to lymph nodes.^120^, ^121^ It is also possible to avoid off‐target effects and decrease autoimmune toxicity by targeted transfection to APCs.^122^ Ugur Sahin's group reported the delivery of RNA‐encoded antigens by using DOTMA/DOPE liposomes (**Figure** **8**). They adjusted the net charge of the liposomes for a targeted delivery of antigens to DCs. The RNA‐loaded liposomes induced strong effector and memory T‐cell responses, and led to IFNα‐dependent rejection of tumors in different animal models. Importantly, a phase I trial showed that a strong antigen‐specific T‐cell response was elicited in three patients with low‐dose liposomes.^123^ Huang and co‐workers developed a lipid/calcium/phosphate (LCP) nanoparticle for the delivery of MUC1 mRNA, a highly expressed tumor‐associated antigen in many cancers. Through surface modification with mannose, LCP NPs successfully released MUC1 mRNA into the cytosol of DCs and induced an MHC I‐restricted cytotoxic T‐cell response. By combining LCP with anti‐CTLA‐4, the mRNA‐loaded LCP NP vaccine induced a strong antigen‐specific immune response in mice bearing triple negative breast cancer.^124^ In order to improve the translational capacity and safety profile, researchers attempted to use nucleoside‐modified mRNA. Compared with unmodified mRNA, nucleoside‐modified mRNA encoding tumor antigens was safer, however it also prevented the induction of type I IFN and limited the activation of APCs. The author demonstrated that the reduction of DC activation could be compensated by codelivery of tumor antigens and a TLR agonist with a DOTAP‐cholesterol lipoplex.^125^

### 2.4 Cell Membrane‐Coated Nanovaccine

Cell membranes maintain many key functions of cells, such as cellular targeting and cell to cell interaction.^126^ Instead of complicated biochemical synthesis, a facile technique to realize cell‐membrane functions is to directly derive and coat natural cell membranes onto a synthetic nanoparticulate core.^127^, ^128^, ^129^ Zhang and co‐workers demonstrated that platelet membrane‐coated nanoparticles showed selective adhesion to damaged vasculature and enhanced binding to platelet‐adhering pathogens.^130^ Leukocyte membrane‐coated nanoparticles can communicate with endothelial cells and transport payloads across inflamed reconstructed endothelium.^131^, ^132^ Among different cell membranes, cancer cell membranes contain a variety of tumor antigens that can be used to develop membrane‐coated nanoparticles as cancer vaccines. For example, a B16‐F10 melanoma cell membrane was successfully coated on a CpG‐loaded PLGA nanoparticle. The cloaked nanoparticle was able to mature dendritic cells in draining lymph nodes with up‐regulation of CD40, CD80, CD86, and MHC‐II. The cancer cell membrane‐coated nanoparticles contained characteristic melanoma antigens such as gp100, tyrosinase‐related protein (TRP‐2) and melan‐A that can increase antigen‐specific T‐cell responses. It was shown that 86% of mice vaccinated with cancer membrane‐coated nanoparticles remained tumor‐free survival for over 150 d, which was more efficient than a mixture of whole B16‐F10 cells and free CpG.^133^ In another example, researchers developed an APC‐targeted nanoparticle by using mannose‐modified cancer cell membranes and TLR7‐loaded PLGA nanoparticles. The nanovaccine led to an enhanced DC activation and was used as a prophylactic vaccine.^134^ Other examples of cell membrane‐coated nanovaccines include erythrocyte membrane‐coated PLGA nanoparticles^135^ and cancer membrane‐coated porous silicon nanoparticles.^136^

Instead of cell membrane‐coated nanoparticles, researchers directly utilized immunogenically dying tumor cells as cancer vaccines. Immunogenic cell death (ICD) is highlighted by the release of tumor antigens and secretion of damage‐associated molecular patterns (DAMPs). The functions of DAMPs include releasing immunostimulatory signals and presenting tumor antigens on DCs for a specific T‐cell response. Moon and co‐workers induced ICD in B16F10 OVA melanoma cells using mitoxantrone. The dying tumor cells expressed HMGB1 ICD markers which were further modified by a CpG‐loaded multilamellar lipid‐polymer hybrid nanodepot. These nanoparticle‐modified dying tumor cells efficiently activated APCs and antigen‐specific CD8+ T cells to elicit a complete tumor regression and durable immune memory in ≈78% of CT26 tumor‐bearing mice.^137^

### 2.5 Inorganic Nanovaccine

**Figure 9:**
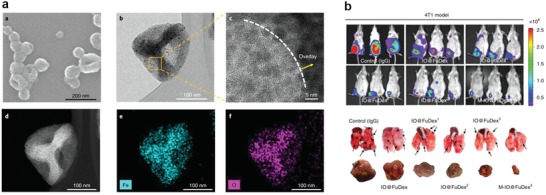
a) Characterization of IO@FuDex. b) Tumor inhibition and antimetastatic capacity of various IO@FuDex formulations in 4T1 tumor model. Reproduced with permission.^141^ Copyright 2018, Nature Publishing Group.

Inorganic nanoparticles are designed to deliver tumor antigens with additional benefits, such as providing imaging contrast for theranostics or causing heat‐induced or reactive oxygen species (ROS)‐induced immunogenic cell death.^138^ Seong and co‐workers developed an iron oxide–zinc oxide core–shell nanoparticle to deliver tumor peptide antigens. The ZnO surface can bind to certain peptide motifs with high binding affinity, while the iron oxide core can provide imaging contrast for monitoring the migration of the nanovaccine as well as the activated DCs with magnetic resonance imaging (MRI).^139^ Magnetic navigation was also used to direct iron oxide nanoparticles to increase tissue specific accumulation and decrease off‐target effects.^140^ Fucoidan‐dextran‐based superparamagnetic iron oxide nanoparticles were synthesized to conjugate anti‐PD‐L1, anti‐CD3, and anti‐CD28 as a cancer vaccine (**Figure** **9**). The nanovaccine can accumulate in tumors under the guidance of an external magnetic field. Compared with free anti‐PD‐L1, the nanoparticles extended the median survival from 32 to 63 d with a dose of less than 1% of free anti‐PD‐L1, and significantly reduced the adverse effects under magnetic navigation.^141^

Gold nanoparticles were used in a nanovaccine to allow for a fast image‐guided prediction for the therapeutic response of immunomodulators. Gold nanoparticles can serve as a contrast agent for CT imaging and enable tracking the accumulation of immunomodulators in tumors. Researchers showed that the tumor uptake of anti‐PD‐L1 conjugated gold nanoparticles significantly inhibited tumor growth. Treatment with these gold nanoparticles required only one fifth of the standard clinical dose to prevent tumor growth.^142^ Gold nanoparticles were also used to induce an immunogenic photothermal therapy. A hydrogel composed of hexapod‐like CpG‐loaded gold nanoparticles was formulated to induce a controlled release of CpG and expression of heat shock protein 70 under laser irradiation at 780 nm. Such formulation led to an increase of tumor‐associated antigen‐specific IgG in serum and an interferon‐γ production from splenocytes.^143^

Upconversion nanoparticles (UCNPs) are also developed to enhance immune responses.^144^ Liu's group simultaneously delivered photosensitizers and TLR agonists with UCNPs. The nanoparticles induced a strong photodynamic therapy (PDT) and caused immunogenic cell death that released a pool of tumor antigens. The released antigens further induced DC maturation and a strong antitumor immune response with the help of delivered TLR agonists.^145^ Similar PDT‐induced immunotherapy was achieved by an enzyme‐encapsulated, photosensitizer‐loaded hollow silica nanoparticle^146^ or a hollow mesoporous MnO_2_ nanoshell containing photosensitizer and doxorubincin.^147^

Other examples of inorganic nanovaccines include using MgAl‐layered double hydroxide (LDH) nanoparticles to codeliver CpG and peptide antigens;^148^ using zinc‐doped iron oxide magnetic nanoparticle‐loaded phospholipid micelles to codeliver poly I:C, TLR3 agonist and OVA peptide antigens;^149^ using lipid‐coated zinc phosphate hybrid nanoparticles to codeliver multiple peptide antigens and TLR 4 agonist;^150^ using liposome‐coated gold nanoparticles with anti‐CD11c targeting ligands to codeliver MPLA adjuvant and TRP2 peptide antigen;^151^ and using multiwalled carbon nanotubes to codeliver CpG, OVA and anti‐CD40.^152^

Current strategies in developing cancer vaccines involve delivering tumor‐associated antigens or neoantigens that are either in the form of proteins, peptides, nuclei acids or cell membranes. The most commonly used delivery systems are made of lipids, proteins, polymers, polysaccharides or inorganic nanoparticles such as gold or iron oxide. The tumor antigens can be either encapsulated into nanoparticles or absorbed onto the surface of “sticky” nanoparticles, or conjugated to delivery materials. The loading process happens either during the assembling of delivery system or in situ in tumors. One of the important advantages of nanodelivery systems is that they can codeliver multiple antigens or codeliver antigens and immune agonists for dendritic cells. Cancer vaccines are often combined with checkpoint blockades for better efficacy (**Table** **1**).

**Table 1:** Representative nanovaccines for tumor treatment

Nanosystem	Immunomodulator	Tumor model	Combination	Ref.
PLGA nanoparticle, DOTAP‐coated PLGA nanoparticle, functionalized PLGA‐PEG nanoparticle	Tumor‐derived protein antigens released during radiotherapy	B16F10 & 4T1	Combined with anti‐PD‐1, increased abscopal effect	88
High‐density lipoprotein‐mimicking nanodisc	Neoantigen peptide, CpG	MC‐38 and B16F10	Combined with anti‐PD‐1, anti‐CTLA‐4	97
Evans blue conjugate that binds to albumin in vivo as nanovaccine	Neoantigen peptide, CpG	MC‐38 and B16F10	Combined with anti‐PD‐1 and/or Abraxane	108
PEG‐*b*‐PC7A copolymer nanoparticle	Antigen peptide	B16F10, MC‐38, TC‐1	Combined with anti‐PD‐1	113
PEI‐absorbed mesoporous silica microrod	Neoantigen peptide, CpG, GM‐CSF	B16F10, TC‐1	Combined with anti‐CTLA‐4	114
Self‐assembled intertwining CpG‐stat3 shRNA nanocapsule, PPT‐*g*‐PEG copolymer	Neoantigen peptide, CpG, stat3 shRNA	MC‐38,	\	115
PLGA‐nanoparticle‐stabilized Pickering emulsion adjuvant system	Tumor‐associated antigen peptide	B16‐MUC1	\	117
DOTMA/DOPE liposome	RNA‐encoded antigens	B16‐OVA, B16F10, CT26, TC‐1, phase I clinical trial	\	123
B16F10 cell membrane‐coated PLGA nanoparticle	Cancer cell membrane, CpG	B16F10	Combined with anti‐PD‐1 and anti‐CTLA‐4	133
Dying tumor cell modified with hyaluronic acid incorporated liposome	Dying tumor cells, CpG	B16F10‐OVA, CT26	Combined with anti‐PD‐1	137
Iron oxide–zinc oxide core–shell nanoparticle	Tumor‐associated antigen peptide	MC38‐CEA	\	139
Superparamagnetic iron oxide nanoparticle, fucoidan and aldehyde‐functionalized dextran	Anti‐PD‐L1, anti‐CD3, anti‐CD28	4T1, CT‐26	\	141
Hexapod‐like CpG‐gold nanoparticle hydrogel	CpG	EG7‐OVA	Combined with photothermal therapy	143
PEG‐grafted poly(maleic anhydride‐alt‐1‐octadecene)‐modified upconversion nanoparticle	Toll‐like‐receptor‐7 agonist (imiquimod)	CT26	Combined with surgery, photodynamic therapy and anti‐CTLA‐4	145

## 3 Nanotechnology to Improve Immune Checkpoint Blockade

### 3.1 Delivery of Checkpoint Inhibitors

To improve the durable response rate of checkpoint blockade therapy, nanotechnology has been used to deliver checkpoint inhibitors.^153^, ^154^ In one study, anti‐PD‐1 peptide (APP) hollow gold nanoshell were coencapsulated in PLGA nanoparticles. They exhibited a slow and continuous release of APP for 40 d. Release was easily accelerated by illumination with a near‐infrared (NIR) laser. The sustained release maintained an effective concentration of APP in the tumor for durable inhibition.^155^ Researchers also developed a CpG‐based nanocarrier for anti‐PD‐1 delivery. Treatment with anti‐PD‐1‐loaded CpG nanoparticles induced a complete response rate in 40% of mice in an incomplete tumor‐resection model. Coadministration of free aPD1 and CpG only showed a limited delay of tumor growth and inefficiency in preventing tumor relapse. The nanoparticles also showed better efficacy in hindering tumor metastasis after surgery than free aPD1 plus CpG.^156^

An engineered cellular nanovesicle presenting PD‐1 receptors was developed to disrupt the PD‐1/PD‐L1 interaction between T cells and tumor cells. Researchers engineered HEK 293T cells to stably express the PD‐1 receptor. The cell membrane was collected and formulated into nanovesicles through the membrane extrusion method.^157^ Compared with free antibodies, the engineered cellular nanovesicles showed prolonged circulation time in blood and induced an enhanced immune response against melanoma cells in vivo. The nanovesicles could load other therapeutics such as IDO inhibitors to overcome immunosuppression. Through binding with PD‐L1 receptors on tumor cells, the nanovesicles were able to decrease the loss of CD8+ T cells and increase their infiltration into tumors.

Researchers also developed a microneedle patch for local delivery of checkpoint inhibitors. The microneedle was made of hyaluronic acid and integrated with pH‐sensitive dextran nanoparticles that encapsulated anti‐PD‐1 and glucose oxidase. The glucose oxidase transforms blood glucose to gluconic acid to induce a self‐dissociation of dextran NPs and anti‐PD1 release. A single administration of the loaded microneedle patch induced a stronger immune response than the microneedle patch without glucose oxidase or direct intratumor injection of free anti‐PD1.^158^

### 3.2 Codelivery of Checkpoint Inhibitors and Costimulatory Immunomodulators

**Figure 10:**
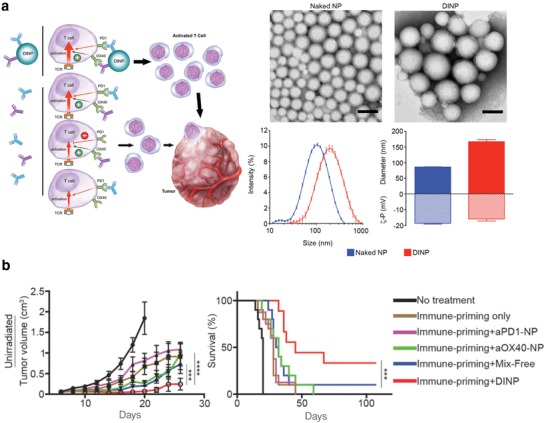
a) Dual immunotherapy nanoparticle (DINP) conjugated with aPD‐1 and aOX40 bind to its target proteins simultaneously. b) DINP improves the efficacy of combination immunotherapy in vivo. Reproduced with permission.^161^ Copyright 2018, Wiley.

Most immunotherapies that directly aim to only stimulate a cytotoxic T‐cell response fail due to immunosuppression in the TME. Additionally, treatment via single immune checkpoint blockade leads to resistance and a limited response rate. Therefore, there is a strong interest to efficiently deliver immunomodulators targeting different inhibitory pathways or both inhibitory and costimulatory pathways.^159^, ^160^ For example, optimal T‐cell activation is achieved when both immunomodulatory agents simultaneously engage T cells and promote synergistic pro‐activation signaling. However, standard administration of multiple therapeutics as free antibodies results in suboptimal T‐cell activation due to only a subset of the T cells binding to both immunomodulators. To improve this T‐cell activation, Wang's group developed a dual immunotherapy nanoparticle (DINP) that spatiotemporally codelivered antagonistic antibodies (anti‐PD‐1) and agonistic antibodies (anti‐OX40) (**Figure** **10**). Compared with a mixture of free anti‐PD‐1 and anti‐OX40, DINP elicited significantly greater number of IFN‐γ producing T cells and higher overall activity of IFN‐γ production. Combination immunotherapy administered in the form of DINP was more effective than the same regimen administered as free antibodies in an immune‐primed B16‐F10 melanoma model and immune‐primed 4T1 breast cancer model. A tumor‐rechallenge experiment showed that 83% of cured mice totally rejected the tumor recurrence, indicating that DINP induced durable antitumor immunological memory. Mechanism studies revealed that DINP treatment provided a significantly higher percentage of T cells that bond to both anti‐PD‐1 and anti‐OX40, when compared with treatment by free antibodies. It was also demonstrated that DINP‐treated mice had a significantly higher number of tumor infiltrating CD8+ T cells, a higher ratio of CD8+ to regulatory T cells, and a higher ratio of effector memory to central memory T cells than the mixture of free antibody‐treated animals.^161^

In another study, an immune‐switch nanoparticle was developed to overcome the immunosuppressive tumor microenvironment by codelivering anti‐PD‐L1 and anti‐41BB. The immune‐switch nanoparticle could switch off the immunosuppressive PD‐L1 pathway on tumor cells, and at the same time switch on the costimulatory 4‐1BB pathway on CD8+ T cells. The nanoparticle also enhanced the interaction between T cells and tumor cells. Compared with soluble antibodies or nanoparticles conjugated with antibodies separately, the immune‐switch nanoparticle significantly delayed tumor growth in melanoma and colon cancer models. Mechanistically, the enhanced tumor growth inhibition was achieved by inducing expansion of a specific population of CD8+ T cells with an altered T‐cell receptor sequence signature.^162^

## 4 Nanotechnology in Adoptive Immunotherapy

Adoptive cell therapy (ACT) is defined as expanding tumor‐specific T cells isolated from a patient's peripheral blood or tumor biopsies ex vivo, culturing with stimuli or genetically engineering the cells, and then infusing them back into patients for cancer treatment.^163^, ^164^, ^165^, ^166^ It is one of the most effective therapies for patients suffering metastatic melanoma with an approximate objective cancer regression rate of 50%.^167^ This ex vivo activation process for tumor‐specific T cells bypasses normal tumor immunosuppression in vivo and avoids systematic autoimmune reactions.^167^, ^168^ However, the objective response rate of ACT in solid tumors remains low. This is due to the immunosuppressive TME deactivating ACT‐produced T cells after infusion. Therefore, persistence of T‐cell activation within the tumor microenvironment is important to improve the overall efficacy of ACT.

Nanoparticles can improve the efficacy of ACT by providing a T‐cell targeting strategy and delivering immunosuppression inhibitors. One such target is TGF‐β, a key immunosuppressive cytokine in the tumor microenvironment. Irvine and co‐workers formulated a TGF‐β inhibitor‐encapsulated PEGylated liposome that targets ACT T cells. They compared two T‐cell targeting strategies via an internalizing receptor CD90 or a noninternalizing receptor CD45. Both of the strategies improved the efficacy of ACT. However, when T cells were preloaded with the liposomes ex vivo and then injected, the CD45 targeting strategy showed greater enhancement of T‐cell activity. By contrast, when the liposomes were administered intravenously in vivo, the CD90 targeting strategy led to greater tumor regression.^169^ Researchers also studied the influence of integrin‐mediated cell adhesion on CD4+ T‐cell activation and proliferation. Researchers formed a PEG hydrogel that cross‐linked with two fibronectin‐derived peptides, cyclic Arg‐Gly‐Asp (cRGD) and cyclic Leu‐Asp‐Val (cLDV), and further decorated it with a quasi‐hexagonal array of gold nanoparticles (AuNPs) functionalized with anti‐CD3. Both of the integrin binding ligands enhanced T‐cell activation, while only cRGD enhanced T‐cell proliferation with memory.^170^ Gold nanoparticles also induced higher siRNA transfection efficiency in cytotoxic T cells to which short laser pulses were applied.^171^

**Figure 11:**
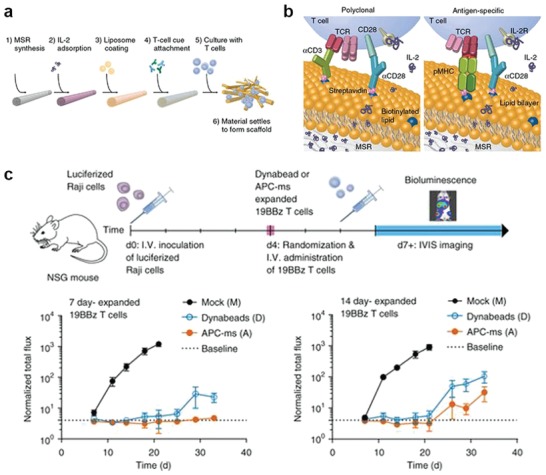
a) Process for preparing APC‐ms from MSRs. b) Schematic showing polyclonal and antigen‐specific T‐cell expansion. c) In vivo efficacy of restimulated 19BBz CAR‐T cells in a disseminated lymphoma xenograft model. Reproduced with permission.^174^ Copyright 2018, Nature Publishing Group.

Synthesized nano‐/microcomplexes can mimic APC's functions and stimulate T cells ex vivo. Efficient tumor‐specific T‐cell expansion requires the interaction between T cells and APCs such as dendritic cells (DCs). However, using autologous DCs to activate T cells ex vivo is both time consuming and expensive. Therefore, APC mimicking nano‐/microplatforms such as magnetic beads or polymeric nanoparticles, are being developed to provide a high local concentration of tumor‐specific antigens and cytokines for durable T‐cell stimulation.^172^, ^173^ A recent study applied supported lipid bilayers (SLBs) to high‐aspect ratio MSRs to act as an APC mimic scaffold. IL‐2, anti‐CD3 and anti‐CD28 were absorbed in the MSRs and acted as stimulatory cues for T‐cell expansion (**Figure** **11**). The APC mimic scaffold promoted two‐ to tenfold greater polyclonal expansion of primary mouse and human T cells compared with commercial expansion beads. The expansion efficiency was highly related to the density of stimulatory cues. By changing anti‐CD3 to pMHC, APC mimic scaffolds enable antigen‐specific expansion of rare cytotoxic T‐cell subpopulations at a greater magnitude than autologous monocyte‐derived dendritic cells. The scaffolds were also used to expand CD19 CAR‐T cells and showed an efficient antitumor effect in a xenograft lymphoma model.^174^ In another study, a carbon nanotube‐polymer nanoparticle complex was developed as an APC mimic platform. Researchers attached pMHC and anti‐CD28 on carbon nanotubes and combined them with IL‐2 and magnetite coloaded PLGA nanoparticles. The APC mimic complex enabled magnetic separation after incubation with T cells. It expanded cytotoxic T cells to a level comparable to clinical standards using only 1/1000th the amount of soluble IL‐2. The expanded T cells inhibited tumor growth efficiently in a murine melanoma model. Furthermore, the complex was able to expand a greater number of human Epstein‐Barr Virus‐specific CD8+ T cells than autologous DCs .^175^

**Figure 12:**
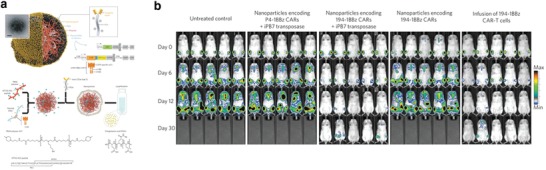
a) Design and manufacture of lymphocyte‐programming nanoparticles. b) Nanoparticle‐programmed CAR lymphocytes can cause tumor regression with efficacies similar to adoptive T‐cell therapy. Reproduced with permission.^178^ Copyright 2017, Nature Publishing Group.

Another application of nanotechnology in adoptive immunotherapy is delivering genome‐editing tools such as CRISPR/Cas9 to achieve in situ CAR‐T therapy.^176^, ^177^ A nanodelivery system could be used to transport leukemia‐specific CAR genes into host T cells within human bodies to avoid the safety issues currently present during the manufacturing process. To achieve targeted gene delivery to T cells in vivo, researchers incorporated an anti‐CD3e f(ab′)2 fragments‐polyglutamic acid (PGA) conjugate to the surface of poly(β‐amino ester) (PBAE) nanoparticles (**Figure** **12**). The PBAE was functionalized with peptides containing microtubule‐associated sequences (MTAS) and nuclear localization signals (NLS) to promote nuclear import of genes by microtubule transport machinery. The targeted nanoparticle was utilized to codeliver a plasmid DNA encoding leukemia‐specific 194‐1BBz CAR and a hyperactive form of transposase (iPB7). The nanoparticle selectively bound to 34% of circulating T cells with a limited binding rate of 5.9% to off‐target cells. The CAR‐transfected circulating T cells increased from 5.8% to 19.7% between day 6 and day 12, while the fraction of transfected phagocytes in liver and spleen gradually decreased. The 194‐1BBz receptors on T cells were detected on day 6 post‐transfection and remained till day 24. Using a B‐cell acute lymphoblastic leukemia mouse model, the researchers showed that reprogramming T cells in situ via nanoparticles achieved similar efficacy in tumor regression, compared with ACT T cells transfected ex vivo by CAR‐encoding viral vectors.^178^ A similar nanotechnology was also used to deliver mRNA for in situ CAR T‐cell manufacture and enhancement.^179^

To avoid immunosuppression of ACT in solid tumors, researchers developed an iRGD‐modified liposome, encapsulating PI3K inhibitors for overcoming immunosuppression and alpha‐GalCer agonists for T‐cell activation. The nanoparticles modified the tumor microenvironment and created a therapeutic window for ACT. Within the therapeutic window, the survival of mice doubled compared with conventional CAR‐T therapy.^180^

**Figure 13:**
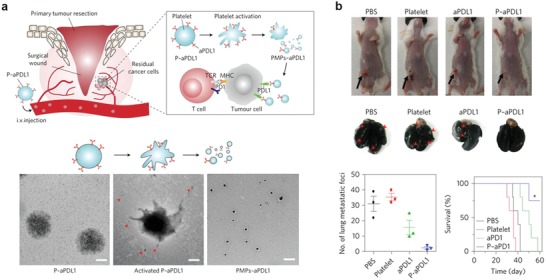
a) In situ activation of P–aPDL1 promoted release of anti‐PDL1 (aPDL1) and cytokines. b) P‐aPDL1 treatment of recurrent triple negative 4T1 tumor. Reproduced with permission.^181^ Copyright 2017, Nature Publishing Group.

Engineered platelets have also attracted interest for cancer immunotherapy due to the ability of platelets to accumulate at inflammatory sites and to recruit and activate T cells via chemokines. Gu and co‐workers engineered platelets by attaching anti‐PD‐L1 on their surface (**Figure** **13**). The engineered platelets were used to generate platelet‐derived microparticles to facilitate binding between anti‐PD‐L1 and tumor cells, which further reduced postsurgical tumor recurrence and metastasis.^181^ The same group conjugated anti‐PD‐1‐decorated platelets to hematopoietic stem cells (HSCs) for the treatment of acute myeloid leukemia. Such strategy takes the advantage of homing capability of HSCs and local release of checkpoint inhibitors by in situ activation of platelets, which significantly suppressed the growth and recurrence of leukemia in mice.^182^

## 5 Other Applications of Nanotechnology in Cancer Immunotherapy

### 5.1 Enhancing Tumor Recognition

**Figure 14:**
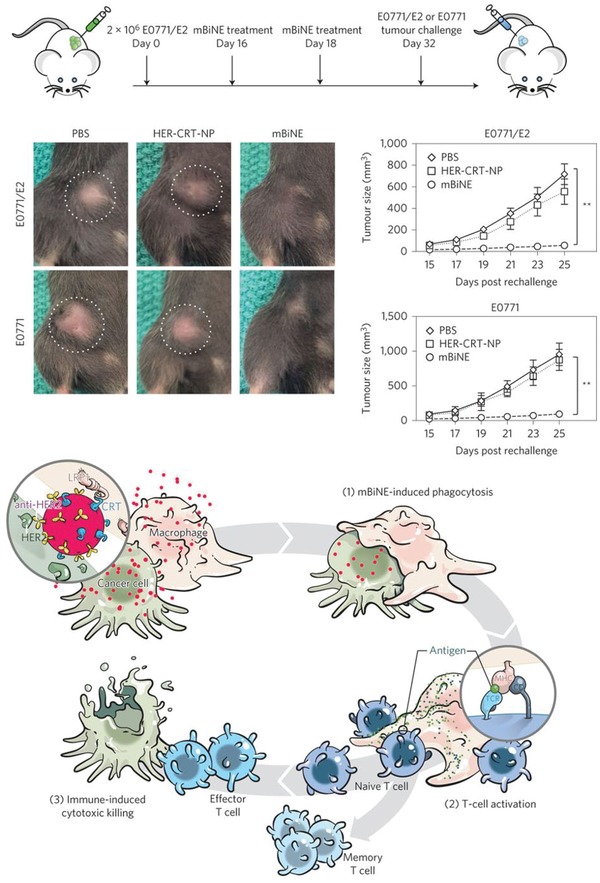
The mBiNE induces systemic, durable antitumor immunity. Reproduced with permission.^183^ Copyright 2017, Nature Publishing Group.

Instead of directly boosting the host's immune system, nanoparticles can facilitate the recognition of cancer cells by immune cells. By introducing an “eat‐me” signal to cancer cells using targeted nanoparticles, immunogenic clearance can be enhanced for a subsequent adaptive immune response. For example, Kim and co‐workers designed a multivalent bi‐specific nanobioconjugate engager (mBiNE) through conjugating a carboxylated polystyrene nanoparticle with human epidermal growth factor receptor 2 (HER2) antibodies and prophagocytic protein calreticulins (CRTs) (**Figure** **14**). The mBiNE elicited cancer cell phagocytosis by macrophages, resulting in a cross‐presentation of tumor antigens on macrophages and a CD8+ T‐cell response. The antitumor effect of mBiNE was confirmed in an HER2 overexpressed tumor model. Interestingly, the tumor re‐challenge experiment showed that mBiNE not only elicited a durable immune memory in HER2 overexpressed tumors but also protected from HER2 negative subclones.^183^

### 5.2 Tuning the Tumor Microenvironment

The tumor microenvironment (TME) plays an important role in interacting with the host's immune system and modifying immune responses.^184^ The TME is a chronic inflammatory and immunosuppressive environment, in which the recruitment of immune cells is mediated by secreted chemokines, and their cytotoxic functions are greatly suppressed by immunoregulatory cells.^185^ A variety of immunosuppressive cells are found in the TME, such as myeloid‐derived suppressor cells (MDSC), regulatory T (Treg) cells and type 2‐polarized macrophages (M2). They regulate anticancer immune responses either via the expression of inhibitory checkpoint molecules or via the production of immunoregulatory cytokines, such as TGF‐β, IL‐10, IL‐4, IL‐13, or IDO.^186^, ^187^ In order to improve cancer immunotherapy, it is important to understand the TME and overcome its immunosuppression. Therefore, different inhibitory agents are studied that target immunosuppressive cells as well as their secreted cytokines in the TME. These agents are meant to restrict the functionality of immunosuppressive cells or reduce the production of immunoregulatory cytokines. Through the EPR effect, nanoparticles can accumulate in tumors and deliver inhibitors, potentially changing the TME. For example, tumors expressing BRAF mutant are highly immunosuppressive and resistant to immune checkpoint therapies. It was determined that the expression of Wnt family member 5A (Wnt5a) plays a key role in controlling the immunosuppressive TME. Huang's group designed a trimeric trap protein that can bind to Wnt5a, and then delivered its correlated plasmid DNA with a cationic lipid‐protamine‐DNA nanoparticle. In order to effectively activate T cells, researchers utilized low‐dose doxorubicin (DOX) to induce an ICD. The combination of DOX‐induced ICD and Wnt5a trapping remodeled the immunosuppressive TME by increasing DC maturation, enhancing the infiltration and activation of cytotoxic T cells, reducing immunosuppressive cells, and increasing immune cytokines such as IL‐12α, TNF‐α and IFN‐γ. Such results indicated that the nanoparticles were able to change the TME from an immunosuppressive Th‐2 phenotype to an immunostimulatory Th‐1 phenotype.^188^ Similarly, a liposome‐protamine‐DNA nanoparticle was generated to codeliver a PD‐L1 trap‐encoded plasmid DNA and oxaliplatin,^189^ or plasmid DNAs encoding for both CXCL12 and PD‐L1 traps.^190^, ^191^ The combination therapies modulated the TME and facilitated tumor‐specific killing.

**Figure 15:**
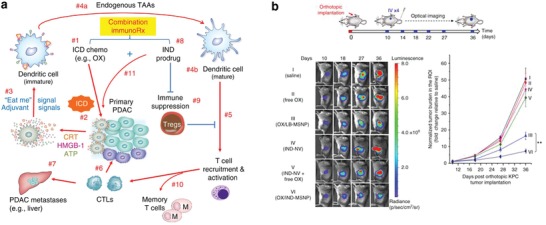
a) Schematic to illustrate how dual delivery of OX and IND may impact the anti‐PDAC immune response. b) Dual delivery of OX plus IND‐NV by MSNP induced effective anti‐PDAC immunity in the orthotopic tumor model. Reproduced with permission.^192^ Copyright 2017, Nature Publishing Group.

Researchers also combined an IDO inhibitor with other immunogenic therapies to overcome immunosuppression in the TME. For example, the immunosuppressive TME of pancreatic cancer was improved by codelivery of an IDO inhibitor and oxaliplatin using a lipid bilayer‐coated mesoporous silica nanoparticle (MSNP) (**Figure** **15**).^192^ By integrating IR780 and IDO inhibitor NLG919 into MPEG‐PCL micelles, Qian and co‐workers effectively suppressed the primary tumor and induced an abscopal effect to inhibit the growth of distal tumors.^193^ Lin and co‐workers developed a chlorine‐based nanoscale metal‐organic framework (MOF) to encapsulate IDO and combined this with immunogenic photodynamic therapy. The singlet oxygen generated by photodynamic therapy combined with nMOF induced ICD and successfully treated primary and distant tumors on CT26 and MC38 animal models.^194^ The same group also reported a coordination polymeric core–shell nanoparticle loaded with oxaliplatin and pyropheophorbide‐lipid conjugates for immunogenic chemo and photodynamic therapy. The multimodality treatment enhanced antitumor immunity and increased the efficacy of PD‐L1 checkpoint blockade therapy.^195^

In addition to simultaneous delivery, nanoparticles can achieve sequentially stimuli‐triggered release in the TME. In a study, researchers formed a peptide self‐assembling nanoparticle composed of a peptide antagonist (PPA‐1) and an IDO inhibitor (NLG919). To achieve a sequentially stimuli‐triggered release, peptides containing 3‐diethylaminopropyl isothiocyanate (DEAP) and matrix metalloproteinase‐2 (MMP‐2) were used for the formulation of micelles. Within the acidic tumor stroma, the reduction of DEAP triggered a breakdown of the micelle core for a burst release of NLG919. A sequential release of PPA‐1 was achieved after the hydrolysis of the peptide substrate by MMP‐2 enzymes.^196^ In another study, researchers delivered an IDO inhibitor (1MT) together with a photosensitizer PpIX peptide by conjugating them to PEG via a caspase‐3‐sensitive DEVD peptide sequence. The PpIX‐1MT peptide is able to self‐assemble into nanoparticles and release 1MT under a caspase‐3 stimuli‐triggered manner. With light irradiation, the nanoparticles induced reactive oxygen species as well as immunogenic cell death that facilitated the expression of caspase‐3 and tumor antigens. The triggered release of IDO inhibitors could then enhance the activation of CD8+ T cells and inhibit tumor growth in mice bearing primary colon tumors or their lung metastases.^197^

Nanoparticles can directly target immunosuppressive cells in the TME. MDSCs play an important role in tumor‐induced immunosuppression. Depletion of MDSCs in tumors provides a promising way to improve the efficacy of cancer immunotherapy. Benoit and co‐workers designed a PEGylated lipid nanocapsule loaded with a lauroyl‐modified gemcitabine, which can be taken up by monocytic cells rather than other immune cells. Low‐dose administration of the lipid nanocapsule reduced tumor‐infiltrating MDSCs and enhanced efficacy of adoptive T‐cell therapy in lymphoma and melanoma‐bearing mice.^198^ Treg cells maintain immune homeostasis and prevent autoimmune diseases in healthy tissues. However, they are highly activated in the TME and downregulate the functions and proliferations of effector T cells. Kim and co‐workers developed a tLyp1 peptide‐conjugated hybrid nanoparticle to target Nrp1 on Treg cells. The expression of Nrp1 on CD4+FoxP3+ Treg cells showed high correlation with poor clinical outcome in patients. Kim and co‐workers used Nrp1‐targeted nanoparticles to deliver imatinib, a Treg‐cell inhibitor blocking stat3 and stat5 pathways, to Tregs and suppress their functions. By combining with CTLA‐4 checkpoint blockade therapy, they boosted T‐cell responses and induced tumor destruction.^199^ An M2‐phenotype tumor‐associated macrophage (TAM) is another immunosuppressive cell that restricts the functions of DCs and CD8+ T cells. Researchers encapsulated IL‐12 into a pH‐sensitive poly(β‐amino ester) nanoparticle to reverse TAMs from M2 to M1 phenotypes.^200^ Additionally, researchers developed dual‐targeting fusion peptide‐functionalized liposomes that targeted both scavenger receptors B type 1 (SR‐B1) and M2. The dual‐targeting nanoparticle was used to deliver cholesterol‐modified CSF‐1R siRNA to M2 TAMs. CSF‐1R was restrictively expressed by TAMs and responsible for their proliferation. Through delivery of CSF‐1R siRNA by dual‐targeting nanoparticles, 52% of M2 TAMs were efficiently targeted and eliminated, resulting in an 87% decrease in tumor size and prolonged survival in a B16 melanoma mouse model.^201^ A similar strategy was developed by Tian and co‐workers who designed M2 TAM‐targeted peptide‐RNAi nanoparticles to silence VEGF mRNA in M2 TAMs.^202^ In addition, carcinoma‐associated fibroblasts (CAFs) are abundant in the TME and induce immunosuppression by releasing VEGF, IL‐6, IL‐10, and TGF‐β, while also preventing contact between cancer cells and cytotoxic T cells. Therapies targeting CAFs with antifibroblast‐activation protein (FAP) were evaluated in the clinic, but showed severe systematic toxicity. Xie and co‐workers used apoferritin as a photosensitizer nanocarrier and conjugated it with an anti‐FAP single chain variable fragment for selective killing of CAFs under localized irradiation. Instead of direct tumor killing, this treatment effectively suppressed CXCL12 secretion, destroyed tumor extracellular matrix, and improved infiltration of CD8+ T cells.^203^

## 6 Summary and Outlook

Cancer immunotherapy is making rapid progress with many preclinical technologies translating into clinical practice. With the same momentum, some nano‐enabled cancer immunotherapy treatments are already under clinical investigation. These include nanoparticle formulations of STING agonists and nanoparticle formulations of mRNA cancer vaccines. Based on the robust preclinical data, there is reason for high enthusiasm for the success of these treatments.

Other preclinical approaches, such as nanoparticle vaccines and nanoparticle therapeutics to modify tumor microenvironment, are also making steady progress toward clinical translation. It is expected that many of these technologies will be under clinical investigation in the next 3–5 years.

Despite this high enthusiasm, there are also challenges. First, the toxicity profiles of nano‐based immunotherapy need to be fully characterized. It is unclear whether the increased immune activation from nanoparticles will also increase autoimmune side effects. And if nanoparticles can trigger more autoimmune reactions, strategies need to be developed to minimize such occurrences. For example, combined PD‐1 and CTLA‐4 blockades significantly improved the median progression‐free survival of monotherapy in patients with metastatic melanoma; at the same time, it increased the incidence of grade 3 or 4 treatment‐related adverse events from 16.3% for aPD‐1 and 27.3% for aCTLA‐4 to 55.0%.^204^ As nanoparticles have shown better activation for dendritic cells and T cells by costimulation of multiple signaling pathways, the translation of nano‐/microapproaches needs careful assessment of their toxicity. In addition to intrinsic effects of nanoparticle on toxicity, it is also important to characterize the toxicity profiles of each type of biomaterial in immunotherapy applications. Although immune activation is desired in cancer immunotherapy, over‐activation can be detrimental. For example, over‐activation of dendritic cells can cause death of these antigen‐presenting cells rather than inducing activation. Lastly, nanotechnology generally increases the complexity and cost of manufacturing and commercialization of treatments. Strategies that can minimize this impact will also greatly facilitate the clinical translation of nano‐based immunotherapy treatments.

The field of nanotechnology‐enabled cancer immunotherapy is still in the early stage of development. As stated, we expect high clinical impact from nano‐enabled technologies in the near term. Such impact will generate enthusiasm and interest from the broader cancer research field for further development of nanomedicine in immunotherapy. As the science matures and as we gain further insight on cancer immunology and cancer biology, we expect nanotechnology to enable many novel approaches for immunotherapy. The progress of cancer immunotherapy is increasing relying on biomedical engineering approaches. We expect nanotechnology to be a key driver of cancer immunotherapy success in the near future.

## Conflict of Interest

The authors declare no conflict of interest.
